# Characteristics of Ground-Glass Nodules Detected by Low-Dose Computed Tomography as a Regular Health Examination Among Chinese Hospital Employees and Their Parents

**DOI:** 10.3389/fonc.2021.661067

**Published:** 2021-04-27

**Authors:** Bihan Ouyang, Maoyuan Li, Li Li, Shaohui Liu, Min Li

**Affiliations:** ^1^ Health Management Center, Xiangya Hospital of Central South University, Changsha, China; ^2^ Department of Respiratory Medicine, National Key Clinical Specialty, Branch of National Clinical Research Center for Respiratory Disease, Xiangya Hospital, Central South University, Changsha, China; ^3^ Xiangya Lung Cancer Center, Xiangya Hospital, Central South University, Changsha, China; ^4^ Clinical Research Center for Respiratory Diseases in Hunan Province, Changsha, China; ^5^ National Clinical Research Center for Geriatric Disorders, Changsha, China

**Keywords:** ground glass nodules, health examination, low-dose computed tomography, genetic, family history

## Abstract

**Introduction:**

Annual LDCT has been offered as a regular examination among many unit staff in China. Along with the wide application of LDCT, more and more ground-glass nodules were found. We focused on characteristics and relationship of ground-glass nodules detected by LDCT as a regular health examination among Chinese hospital employees and their parents.

**Methods:**

We recorded LDCT-detected ground-glass nodules (GGNs) in the hospital employees and parents between 2019 and 2020. Clinical information, including age, gender, smoking status was collected and analyzed.

**Results:**

A total of 5,574 employees and 2,686 employs’ parents ≥60 years in Xiangya hospital performed annual physical examination. In total, LDCT incidentally detected ground-glass nodules 392 (24.78%, 392/1,582) in hospital employees and 254 in parents (10.80%, 254/2,352). The GGN-detection rate was significantly greater in employee group than parent group and more non-smokers in former (P <0.001). The detection rate was significantly greater in female than male both in employees group and parents group, and the proportion of female was bigger in employees group (P <0.001). There were more pure-GGNs both in employees group and parents group. There were less participants with solitary GGN in employee group than parent group (P = 0.033). Besides, there were more large GGNs (≥10 mm) (P <0.001), LU-RADS 4 GGNs (P <0.001) and LU-RADS 4B GGNs (P = 0.003), LU-RADS 4C-5 GGNs (P = 0.001) in parent group than employee group. There were 36 employee–parent pairs (27.07%) both had GGNs among 133 pairs who both performed LDCT. GGNs in employees were smaller and lower-grade than their parents (P < 0.001, P = 0.001).

**Conclusions:**

Among the employees and parents who had ground glass nodules, 1/4 of them both detected GGNs. Although the detection rate of GGNs in the parent group was lower than that in the employee group, the grade of nodules was significantly higher. All these suggest that the occurrence and development of ground glass nodules may be related to genetic factors.

## Introduction

In recent years, annual LDCT has been applied for early lung cancer screening worldwide, especially in China. LDCT has been offered as a regular examination among many unit staff among which many are not eligible high-risk participants according to NLST. Along with the wide application of LDCT, more and more ground-glass nodules were found. The International Early Lung Cancer Action Program reported that nonsolid and part-solid nodules were found in 9.2% of 57,496 baseline screenings (1, 2). Zhang et al. (3) retrospectively analyzed LDCT screening among 15,686 Chinese hospital employees and found that 95.5% of patients with screening-detected lung cancer presented as GGO nodules on CT scans. As family history is the main risk factor for lung cancer, the present study focused on characteristics and relationship of ground-glass nodules detected by low-dose computed tomography as a regular health examination among Chinese hospital employees and their parents.

## Methods

### Participants and CT Scans

LDCT was performed as a part of regular health examination in staff above 40-year-old of the Xiangya Hospital and staff’s parents above 60-year-old between 2019 and 2020. The employees below 40-years-old were offered with X-ray but some of them changed for LDCT at their own expense. In general, all the participants were volunteered to take the test. Revolution CT (GE Medical Systems) was used in the examination with 1.3 mm slice thickness, 1mm slice spacing, 100 kV tube voltage, 40–100 mA tube current.

### Clinical Data

LDCT-detected ground-glass nodules (GGNS) in hospital employees and parents were recorded. Clinical information, including age, gender, smoking status was collected and analyzed.

### Nodule Measurements

Fleischner Society defined “a nodule appears as a rounded or irregular opacity, well or poorly defined, measuring up to 3 cm in diameter” and ground-glass nodule (GGN) manifests as hazy increased attenuation in the lung that does not obliterate the bronchial and vascular margins (4). GGNs include pure ground-glass and part-solid nodule. Pure ground-glass nodule has no solid components. A part-solid nodule consists of both ground-glass and solid soft-tissue attenuation components. ALL information of nodules were recorded mainly from CT reports by a radiologist, if there were any ambiguous issues about the nodules, the radiologist would check the CT images. Nodules were measured in long- and short-axi length or diameter. Nodules were classified by The Lung Reporting and Data System (LU-RADS) categories (5).

### Statistical Analysis

We used the Pearson χ^2^ test to compare the GGNs detection rate, and paired-t test to compare the characteristics of GGNs in employee–parent pairs. Statistical analysis was performed in SPSS 23.0.

## Results

### Characteristics of Hospital Employees and CT-Detected GGN

**Figure 1:**
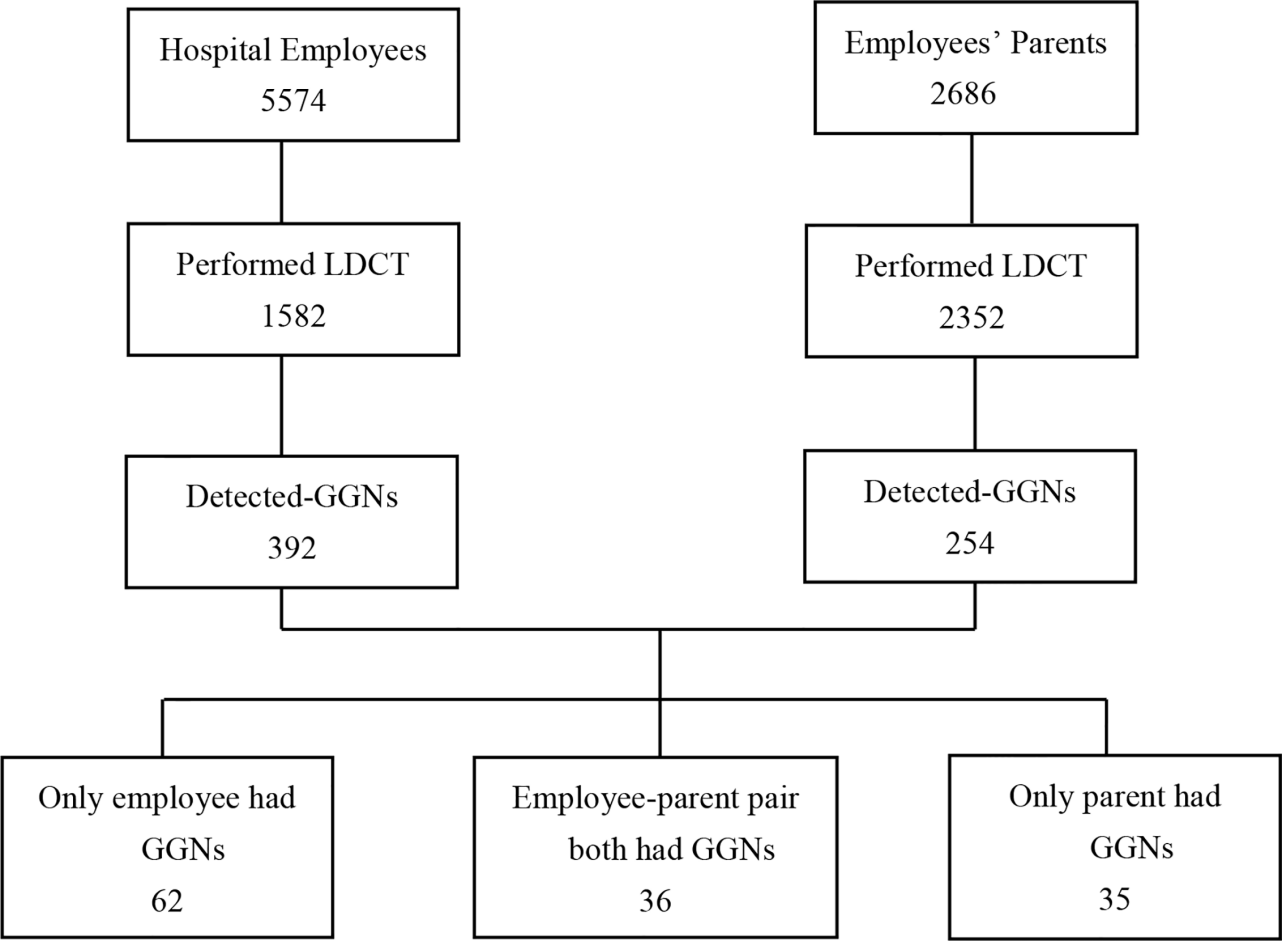
Flowchart of the study analyzed the hospital employees and employee’ parents performing LDCT as regular examination. LDCT, low-dose computed tomography; GGNs, ground-glass nodules.

There were a total of 5,574 employees in Xiangya hospital, among them female employees were 4,224 (75.78%), male employees were 1,350 (24.22%). The overall LDCT participation rate was 28.38% (1,582/5,574), among them female participation rate was 25.33% (1,070/4,224), male participation rate was 37.93% (512/1,350). The percentages of patients performed CT <40 years, 40 to 60 years, and >60 years were 3.49% (120/3,442), 66.32% (961/1,449), 73.35% (501/683), respectively (**Table 1**, **Figure 1**).

**Table 1:** The proportion of employees performing LDCT and which with GGNs according to sex and age.

Characteristics	No. of employees	No. of employees performed LDCT	Rate of employees performed LDCT (%)	CT-detected GGNsN, Rate (%)	*P* value
Total detection rate	5,574	1,582	28.38	392 (24.78)	
Sex					0.002
female	4,224	1,070	25.33	290 (27.10)	
male	1,350	512	37.93	102 (19.92)	
Age					0.143
<40	3,442	120	3.49	22 (18.33)	
40–60	1,449	961	66.32	251 (26.12)	
>60	683	501	73.35	119 (23.75)	

In total, LDCT incidentally detected ground-glass nodules 392 (24.78%, 392/1,582) in hospital employees. Among employees with GGN, 290 (27.10%, 290/1,070) were female and 102 (19.92%, 102/512) were male; the detection rate was significantly greater in female than male (27.10% vs 19.92%, P = 0.002). Among employees with GGNs, 349 (89.02%) participants were non-smokers. The GGN-detection rate in employees age <40 years, 41 to 60 years, and >60 years were 18.33% (22/120), 26.12% (251/961), and 23.75% (119/501), respectively. 68.62% (269/392) had solitary GGN and 31.38% (123/392) had multiple GGNs. There were more employees with pure-GGN than employees with mixed-GGN (331 vs 61). The percentage of GGN <5 mm, 5–9 mm, and ≥10 mm was 27.30% (107/392), 63.77% (250/392), and 8.93% (35/392), respectively. According to the LU-RADS classification, there were 113 (28.83%) employees had LU-RADS 2 GGNs, 246 (62.75%) had LU-RADS 3 GGNs, and 32 (8.42%) had LU-RADS 4 GGNs. Among them, there were 11 nodules were LU-RADS 4B and 14 were LU-RADS 4C or 5 (**Table 2**, **Figure 1**).

**Table 2:** Comparing characteristics of GGNs in employees with GGNs in parents.

Characteristics	GGNs of employees N, Rate (%)	GGNs of parents N, Rate (%)	*P* value
Total detection rate	392/1,582	254/2,352	<0.001
	(24.78)	(10.80)	
Gendar			<0.001
female	290 (73.98)	147 (57.87)	
male	102 (26.02)	107 (42.13)	
Smoking status			<0.001
non-smoker	349 (89.02)	195 (76.72)	
smoker	43 (10.98)	59 (23.28)	
Numbers			0.033
solitary	269(68.62)	194 (76.38)	
multiple	123(31.38)	60 (23.62)	
Density			0.949
pure GGNs	331 (84.44)	214 (84.25)	
part-solid nodules	61 (15.56)	40 (15.75)	
Size (mm)			
<5	107 (27.30)	45 (17.72)	
5–9	250 (63.77)	145 (57.09)	
≥10	35 (8.93)	64 (25.19)	<0.001
LU-RADS			
category 2	113(28.83)	52 (20.47)	
category 3	246(62.75)	150 (59.06)	
category 4	32 (8.16)	49 (19.29)	<0.001
category 4B	11 (2.81)	20 (7.87)	0.003
category 4C-5	14 (3.57)	25 (9.84)	0.001

### Characteristics of Hospital Employees’ Parents and CT-Detected GGN

In total, there were 2,686 employees’ parents ≥60 years participated the regular examination, the average age of parents was 67.85 ± 6.18, among them 1,338 were female and 1,348 were male. The overall participation rate of LDCT was 87.57% (2,352/2,686), female and male participation rate was 86.62% (1,159/1,338), 88.50% (1,193/1,348), respectively.

The overall GGN-detection rate was 10.80% (254/2,352), among them female was 12.68% (147/1,159), male was 8.97% (107/1,193), the detection rate was significantly greater in female than male (12.68% vs 8.97%, *P* = 0.004). Among parents with GGNs, 195 participants were non-smokers. There were more parents with pure-GGN than those with mixed-GGN (214 vs 40), and more with solitary GGN other than multiple GGNs (194 vs 60). The percentages of GGNs <5 mm, 5–9 mm, and ≥10 mm was 17.72% (45/254), 57.09% (145/254), and 25.19% (64/254), respectively. According to the LU-RADS classification, there were 52 (20.47%) parents had LU-RADS 2 GGNs, 150 (59.06%) had LU-RADS 3 GGNs, and 49 (19.29%) had LU-RADS 4 GGNs among them 20 were 4B, 25 were 4C or 5 (**Table 2**, **Figure 1**).

### Comparation and Correlation Analysis of GGNs in Hospital Employees and Their Parents

In total, the GGN-detection rate was significantly greater in employee group than parent group (24.78% vs 10.80%, *P <*0.001). The detection rate was significantly greater in female than male both in employees group and parents group, and the proportion of female was bigger than that in parents’ group (*P <*0.001). There were more non-smokers in employees than in parents’ group (*P <*0.001). There were more pure-GGNs both in employees group and parents group. There were less participants with solitary GGN in employee group than parent group (68.62% vs 76.38%, P = 0.033). Besides, there were more large GGNs (≥10 mm) (25.19% vs 8.93%, P <0.001), LU-RADS 4 GGNs (19.29% vs 8.16%, P <0.001) and LU-RADS 4B GGNs (7.87% vs 2.81%, P = 0.003), LU-RADS 4C-5 GGNs (9.84% vs 3.57%, P = 0.001) in parents’ group than employees group (**Table 2**).

**Figure 2:**
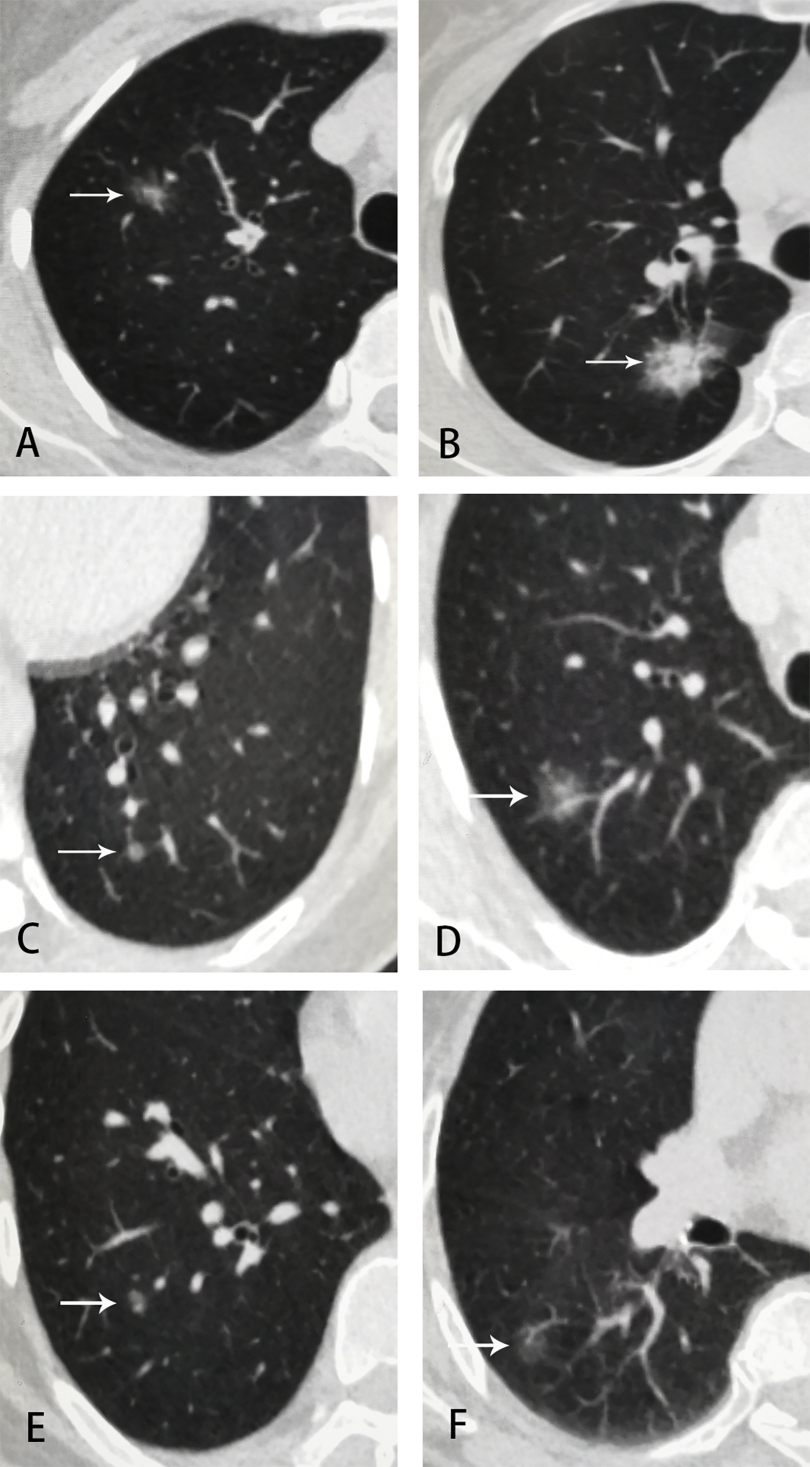
Examples of GGNs in employee-parent pair groups. **(A)** employee 1, female, 42 y, part-solid nodule at right upper lobe, 15 × 10 mm, LU-RADS 4C; **(B)** employee 1’s mother, 67 y, part-solid nodule at right upper lobe, 25 × 18 mm, LU-RADS 5; **(C)** employee 2, female, 51 y, pure-GGN at left lower lobe, 5 mm, LU-RADS 3; **(D)** employee 2’s mother, 73 y, part-solid nodule at right upper lobe, 13mm, LU-RADS 4C; **(E)** employee 3, male, 51 y, pure-GGN at right lower lobe, 6 mm, LU-RADS 3; **(F)** employee 3’s mother, 79 y, pure-GGN at right lower lobe, 14 × 9 mm, LU-RADS 4B.

There were 36 pairs (27.07%) had GGNs, among 133 pairs of employees and their mother/father both performed LDCT. There were more female than male employees in those pairs (75%). GGNs in employees were smaller than their parents (6.11 ± 0.62 vs 11.42 ± 1.38, F = 35, P <0.001), and the LU-RADS categories were lower in employees than their parents (2.64 ± 0.11 vs 3.22 ± 0.14, F = 35, P = 0.001). The rate of multiple GGNs was 36.11% in employees and 33.33% in parents among the pairs. However, there was no significant difference in the density of nodules in employees and their parents, neither in gender (**Table 3**, **Figures 1**, **2**).

**Table 3:** Comparing age, gender, and characteristics of GGNs among three groups in 133 pairs of employees and their parent both performed CT.

Characteristics	GGNs of employee-only group N (%)	Employee-parent pair group				GGNs of parent-only group N (%)
		GGNs of employees N (%)		GGNs of parents N (%)		
Age	46.37 ± 0.63	45.47 ± 0.74		73.22 ± 0.95		69.80 ± 0.94
*P*	0.372		/		0.012	
Gender						
female	50 (80.65)	27 (75.00)		19 (52.78)		26 (74.29)
male	12 (19.35)	9 (25.00)		17 (47.22)		9 (25.71)
*P*	0.511		0.05		0.06	
Numbers						
solitary	45 (72.58)	23 (63.89)		24 (66.67)		22 (62.86)
multiple	17 (27.42)	13 (36.11)		12 (33.33)		13 (37.14)
*P*	0.368		0.804		0.737	
Density						
pure GGN	57 (91.94)	31 (86.11)		29 (80.56)		30 (85.71)
part-solid nodules	5 (8.06)	5 (13.89)		7 (19.44)		5 (14.29)
*P*	0.358		0.527		0.562	
Size (mm)						
<5	22 (35.48)	10 (27.78)		5 (13.89)		6 (17.14)
5–9	39 (62.90)	24 (66.67)		17 (47.22)		23 (65.72)
≥10	1 (1.62)	2 (5.55)		14 (38.89)		6 (17.14)
*P*	0.109		<0.001		0.042	
LU-RADS						
category 2	22 (35.48)	16 (44.45)		7 (19.44)		6 (17.14)
category 3	38 (61.29)	17 (47.22)		16 (44.44)		25 (71.43)
category 4	2 (3.23)	3 (8.33)		11 (30.56)		4 (11.43)
category 4B	1(1.61)	1(2.78)		3(8.33)		3(8.57)
category 4C-5	0	1(2.78)		8(22.22)		1(2.86)
*P*	0.75		0.001		0.015	
Total	62 (100)	36 (100)		36 (100)		35 (100)

Sixty-two employees-only had GGNs but their parent did not. Among them, 80.65%(50/62)were female, 91.94% (57/62) had pure-GGN, and 72.58% (45/62) were solitary GGN. However, only 1 of 62 (1.6%) had GGN ≥10 mm and 2 of 62 (3.2%) were classified as LU-RADS 4. Compared these 62 employees with those employees in 36 employee–parent pairs group, there were no significant difference in the age, gender, and density, number, size, LU-RADS category of GGNs (*P >*0.05) (**Table 3**, **Figure 1**).

Thirty-five parents-only had GGNs but their son/daughter did not. Among them, 74.29% (26/35) were mothers, 85.71% (30/35) had pure-GGN and 62.86% (22/35) were solitary GGN. However, 6 of 35 (17.14%) had GGNS ≥10 mm, and 4 of 35 (11.43%) were classified as LU-RADS 4. Compared these 35 parents with those parents in 36 employee–parent pairs group, parents were older (*P* = 0.012) in latter group and there were more large GGNs (*P* = 0.042) and LU-RADS 4-5 nodules (*P* = 0.015) (**Table 3**, **Figure 1**).

## Discussion

Due to the application of LDCT screening for early stage lung cancer, the number of lung cancer appeared as GGO or GGN is increasing. GGO/GGN is a non-specific radiologic finding, can caused by inflammation or neoplastic proliferation and so on. In Asia, GGO nodules were detected in 7.5% of 2,255 asymptomatic Korean adults (6), and 28.69% of 1,279 LDCT positive participants in a screening program in Taiwan China (7). In our study, the proportion of GGN detected using screening CT was 24.78% in employees group and 10.80% in parents’ group. The different detection rates among studies are probably due to different calculation methods, population ages and performing methods. In the study in Korean, participants were above 45 years old and CT machines with 5-mm thickness with 4-mm intervals were used. However, in the study in Taiwan participants were 19–86 years old and nodule >4 mm in diameter was identified as positive, all CT scans were performed on thin-slice (0.625 mm) machines.

As the age of participants increasing, Zhang et al. showed the lung cancer detection rate were increasing gradually in the “age ≤40 years”, “40 < age ≤ 55 years” and “age >55 years” group, respectively (2). However, we found no significantly increasing of GGN-detection rate as age increasing, the most significant rate was in group “41 to 60 years”, but there were more large, high-grade nodules representing high risk of malignancy in the older-group. Our findings showed age was a key factor for risk of malignance consistent with Zhang’s study. Besides, Li et al. found the average age at diagnosis had significantly decreased from 66.40 to 59.06 in the past two decades using a cumulative meta-analysis (8).

Many authoritative guidelines proposed heavy smoking history as the key factor for risk assessment of lung cancer, that meant most female would not be eligible for lung cancer screening. However, more and more studies showed the proportion of female non-smokers with lung cancer was increasing in recent years, especially in East Asia. She et al. found 587 (65%) of 898 cases with solitary pure GGNs pathologically confirmed as lung adenocarcinoma were female in Shanghai, China (9). Hattori et al. from Japan evaluated 616 surgically resected clinical N0M0 non-small cell lung cancers and found the rate of female was 62% (10). A study in Korea showed 162 (56%) female of 288 patients (non-smoker 68.1%) with lung adenocarcinoma proven by surgery and which appeared as GGNs (11). In our study, the detection rate was significantly greater in female than male either in employee group (non-smokers, 89.02%) or parent group (non-smokers, 76.72%), and in each age group as well. Our study was completely consistent with these researches, as for reasons of these findings are still ambiguous. It may be related to genetic susceptibility (such as EGFR high mutation rate) (12), estrogen and receptors (13), air pollution indoor (such as second-hand smoking or cooking) (14, 15), and history of lung diseases, however further investigation is need to be conducted. As for non-smokers, especially female in Asian, it’s worthy to determine high-risk factors for lung cancer about them based on those possible causes above. Maybe there should be a new adjusted screening criterion for this population, so as to achieve early effective diagnosis and treatment of lung cancer.

In our study, there were 27% employee–parent pair had GGNs at the same time. This result reminded us GGNs may be related to genetic factors, family environment and living habits as well. In recent decades, many researches showed consistently that family history is important etiology for cancers especially lung cancer. Ooi et al. (16) found that the risk of lung cancer in patients with a family history of lung cancer was 2.4 times compared with those without family history. Guo et al. (17) showed the risk of lung cancer in the first-degree relatives of lung cancer patients was about seven times higher than that of healthy people. However, there are a few studies on genetics, family history and living habits about GGNs currently. Because most GGNs remain on follow and few participants have received resection, we have no more genetic information between them to date.

As for lung cancer, genetics had been proved to be a key etiology, but it remains uncertain about family history and living habits. Therefore, the potential relevance of employee-parent pairs is worthy for us to follow on.

Furthermore, former studies discovered first-degree female relatives was a stronger predictor than first-degree male relatives for lung cancer, and a first degree family member with cancer diagnosis before age 50 were associated with increased lung cancer risk, especially among never smokers (18–21). In our research, there were more female (75%) than male employees detected GGNs when their parents had GGNs too, and all of them were non-smokers. It may indicate that female non-smokers are more likely to obtain genetic susceptibility of GGNs from parents. In addition, we found GGNs in employees were less and lower-grade than nodules in their parents among the 36 employee–parent pairs. We speculated nodules in employees would be at earlier stage and grow into nodules like their parents’ when they get old. We also found GGNs of parents in employee-parent pair group were larger and higher-grade than GGNs of parent-only. Whether it suggested that larger and more suspicious nodules were more likely to be passed on to offspring need further research.

## Conclusion

Our study retrospectively analyzed LDCT-detected GGNs among employees and parents. Among the employees and parents who had ground glass nodules, 1/4 of them both detected GGNs. Although the detection rate of GGNs in the parent group was lower than that in the employee group, the grade of nodules was significantly higher. All these suggest that the occurrence and development of ground glass nodules may be related to genetic factors.

## Data Availability Statement

The original contributions presented in the study are included in the article/supplementary material. Further inquiries can be directed to the corresponding author.

## Ethics Statement

The studies involving human participants were reviewed and approved by the Centre for Medical Ethics of Xiangya Medical College of Central South University. The patients/participants provided their written informed consent to participate in this study.

## Author Contributions

BO mainly finished the research data processing, chart production, and article writing. MaL, LL, and SL helped collect clinical information. MiL mainly designed the research, participated in the discussion about the results, and revised the draft. All authors contributed to the article and approved the submitted version.

## Conflict of Interest

The authors declare that the research was conducted in the absence of any commercial or financial relationships that could be construed as a potential conflict of interest.

The handling editor declared a shared affiliation with with several of the authors MaL, LL, SL, and MiL at time of review.
